# Distinct origins of dura mater graft-associated Creutzfeldt-Jakob disease: past and future problems

**DOI:** 10.1186/2051-5960-2-32

**Published:** 2014-03-31

**Authors:** Atsushi Kobayashi, Yuichi Matsuura, Shirou Mohri, Tetsuyuki Kitamoto

**Affiliations:** 1 Department of Neurological Science, Tohoku University Graduate School of Medicine, 2-1 Seiryo-machi, Aoba-ku, Sendai 980-8575, Japan; 2 Influenza and Prion Disease Research Center, National Institute of Animal Health, Tsukuba, Ibaraki 305-0856, Japan

**Keywords:** Creutzfeldt-Jakob disease, Prion protein, Dura mater grafts, Humanized knock-in mouse

## Abstract

Dura mater graft-associated Creutzfeldt-Jakob disease (dCJD) can be divided into two subgroups that exhibit distinct clinical and neuropathological features, with the majority represented by a non-plaque-type of dCJD (np-dCJD) and the minority by a plaque-type of dCJD (p-dCJD). The two distinct phenotypes of dCJD had been considered to be unrelated to the genotype (methionine, M or valine, V) at polymorphic codon 129 of the *PRNP* gene or type (type 1 or type 2) of abnormal isoform of prion protein (PrP^Sc^) in the brain, while these are major determinants of clinicopathological phenotypes of sporadic CJD (sCJD). The reason for the existence of two distinct subgroups in dCJD had remained elusive. Recent progress in research of the pathogenesis of dCJD has revealed that two distinct subgroups of dCJD are caused by infection with different PrP^Sc^ strains from sCJD, i.e., np-dCJD caused by infection with sCJD-MM1/MV1, and p-dCJD caused by infection with sCJD-VV2 or -MV2. These studies have also revealed previously unrecognized problems as follows: (i) the numbers of p-dCJD patients may increase in the future, (ii) the potential risks of secondary infection from dCJD, particularly from p-dCJD, may be considerable, and (iii) the effectiveness of the current PrP^Sc^ decontamination procedures against the PrP^Sc^ from p-dCJD is uncertain. To prevent secondary infection from p-dCJD, the establishment of effective decontamination procedures is an urgent issue. In this review, we summarize the past and future problems surrounding dCJD.

## Introduction

Dura mater grafts used to repair the dural defects at neurosurgery can cause fatal disease years to decades later. The tragedy of dura mater graft-associated Creutzfeldt-Jakob disease (dCJD) was considered to be nearly over. However, recent progress in research of the pathogenesis of dCJD has revealed previously unrecognized problems. In this review, we summarize the past and future problems surrounding dCJD.

Creutzfeldt-Jakob disease (CJD) is a lethal transmissible neurodegenerative disease. The central event in the pathogenesis of CJD is a conformational change of the normal cellular isoform of prion protein (PrP^C^) into an abnormal infectious isoform of prion protein (PrP^Sc^) [1]. The conformational change of PrP^C^ can occur due to either one of three causes: spontaneous conversion in sporadic CJD (sCJD), mutations in the *PRNP* gene in genetic CJD, or infection with PrP^Sc^ in iatrogenic CJD and variant CJD.

One of the most frequent sources of iatrogenic PrP^Sc^ infection is dura mater grafts obtained from human cadavers undiagnosed as CJD. The sum of dCJD (228 cases) and growth hormone-associated CJD (226 cases) accounts for 97% of total iatrogenic CJD cases [2]. A single brand of dura mater graft, Lyodura®, was used for all the dCJD cases in whom the brand name was identified. Although the causative dura mater grafts were manufactured by a German company, 62% (142 cases) of total dCJD cases have been found in Japan [2,3]. Persistent efforts of a Japanese CJD surveillance team have clarified the outline of dCJD outbreaks. The onset of Japanese dCJD patients peaked in the late 1990s, and most of the patients had received the grafts during 1983–1987, while as many as 100,000 persons received the Lyodura® grafts during this period [4,5]. In the process of conducting this elaborate survey, a puzzling mystery about dCJD emerged.

### A mystery about dCJD

**Figure 1:**
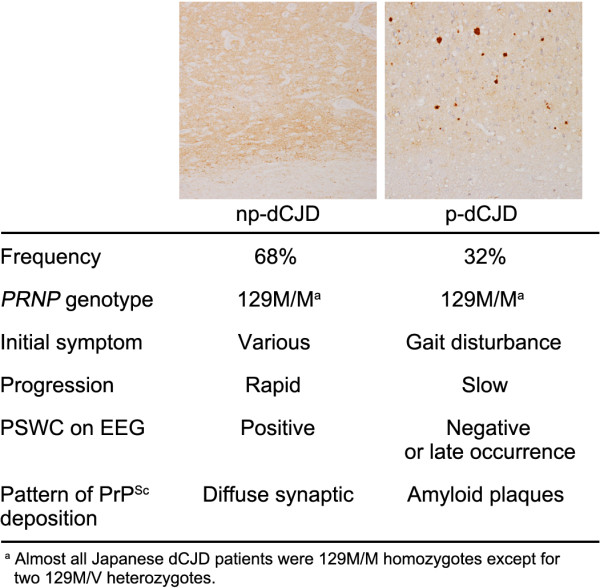
**Clinicopathological features of the two subgroups of dCJD.** The patients with dCJD can be divided into two subgroups, with the majority represented by a non-plaque-type of dCJD (np-dCJD) and the minority by a plaque-type of dCJD (p-dCJD) [11,12]. Neuropathological hallmark of p-dCJD is widespread PrP^Sc^ amyloid plaques, while np-dCJD shows diffuse synaptic-type PrP^Sc^ deposition.

There is growing evidence that dCJD can be divided into two subgroups that exhibit distinct clinical and neuropathological phenotypes, with the majority (68%) represented by a non-plaque-type of dCJD (np-dCJD) and the minority (32%) by a plaque-type of dCJD (p-dCJD) (Figure 1) [6-12]. The clinicopathological features of np-dCJD are identical to those of classical sCJD, whereas p-dCJD is characterized by (i) ataxic gait as an initial symptom, (ii) slow progression of neurological symptoms, (iii) absence or late occurrence of periodic sharp-wave complexes (PSWC) on electroencephalogram (EEG), and (iv) widespread PrP^Sc^ amyloid plaques in the brain [11-16]. There is no significant difference in gender composition, site of graft, age at grafting, and year of grafting between the two subgroups [11,12].

**Figure 2:**
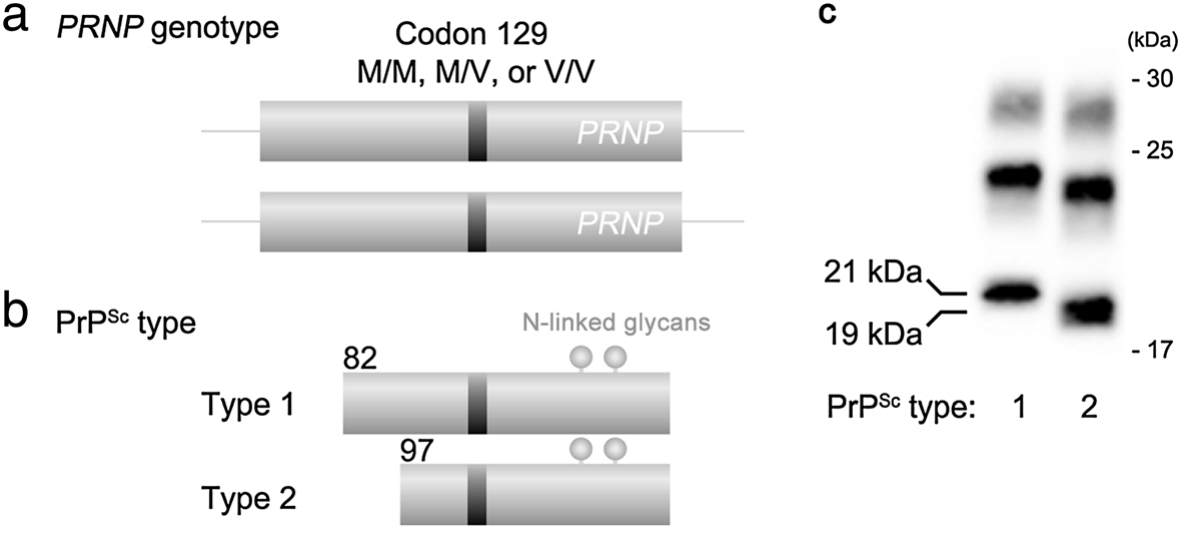
**Two major determinants of the phenotypic heterogeneity of sCJD. (a)** The *PRNP* genotype at polymorphic codon 129. **(b)** Type of PrP^Sc^ in the brain. Types 1 and 2 PrP^Sc^ are cleaved by proteinase K at different sites (at residues 82 and 97, respectively) [19]. **(c)** In western blot analysis, types 1 and 2 PrP^Sc^ are distinguishable by the size of the proteinase K-resistant core of the unglycosylated PrP^Sc^ (21 and 19 kDa, respectively) [17].

sCJD also shows wide phenotypic heterogeneity, and its clinicopathological phenotypes are determined by the genotype at polymorphic codon 129 of the *PRNP* gene and type of PrP^Sc^ in the brain (Figure 2) [17,18]. The codon 129 of the *PRNP* gene shows methionine (M)/valine (V) polymorphism. Two types of PrP^Sc^ (type 1 and type 2) are distinguishable according to the size of the proteinase K-resistant core of unglycosylated PrP^Sc^ (21 and 19 kDa, respectively), reflecting differences in the proteinase K-cleavage site (at residues 82 and 97, respectively) [19]. On the other hand, the two distinct phenotypes of dCJD had been considered to be unrelated to their *PRNP* genotype or type of PrP^Sc^ in the brain [11]. In Japan, almost all dCJD patients had the same genotype, i.e., homozygous for methionine at codon 129 (129 M/M), except two heterozygotes [2], and the type of PrP^Sc^ in their brains had been reported as type 1 [6,10,11]. The reason for the existence of two distinct subgroups in dCJD had remained elusive.

### Solving the mystery

**Figure 3:**
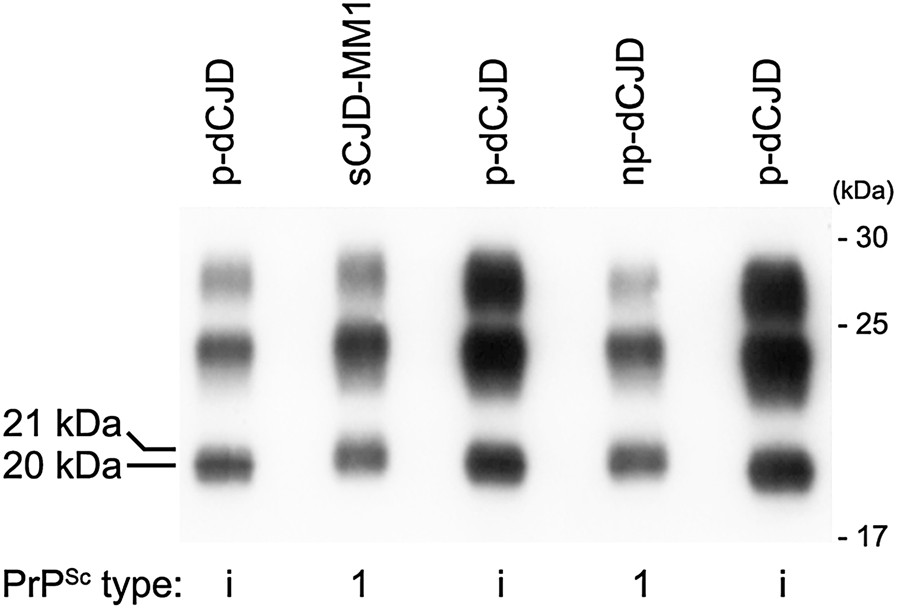
**Distinct biochemical properties of PrP**^**Sc **^**in the two subgroups of dCJD.** The intermediate-sized PrP^Sc^ in p-dCJD, designated as type i PrP^Sc^, was smaller than type 1 PrP^Sc^ in np-dCJD [20].

In 2003, an unusual p-dCJD case was reported [9]. This patient showed the accumulation of unusual PrP^Sc^ with intermediate electrophoretic mobility between types 1 and 2 PrP^Sc^. Then, we reevaluated the biochemical properties of PrP^Sc^ in the two subgroups of dCJD and found that the size of PrP^Sc^ from p-dCJD was invariably smaller than that of type 1 PrP^Sc^ from np-dCJD (Figure 3) [20]. This intermediate-sized PrP^Sc^ was designated as type i PrP^Sc^.

To resolve the mystery of the existence of two distinct subgroups in dCJD, we hypothesized that they might be caused by infection with different PrP^Sc^ strains from distinct sCJD subgroups. According to the *PRNP* genotype and type of PrP^Sc^ in the brain, sCJD is classified into six subgroups (MM1, MV1, VV1, MM2, MV2, or VV2) [17]. MM1 and MV1, which are the predominant subgroups in sCJD, show the same clinicopathological features. Meanwhile, MM2 can be divided into three subgroups based on histopathological criteria (MM2T, thalamic form showing characteristic atrophy of thalamic and inferior olivary nuclei; MM2C, cortical form showing a predominant cortical pathology; or MM2T + C, mixed form) [17,21]. In addition, MV2 is also divided into three subgroups based on histopathological criteria (MV2K showing kuru type PrP^Sc^ amyloid plaques, MV2C showing a predominant cortical pathology, or MV2K + C showing mixed histopathology) [17,18]. The clinicopathological features of np-dCJD, such as short duration of illness, PSWC on EEG, or diffuse synaptic-type PrP^Sc^ deposition in the brain, are identical to those of sCJD-MM1/MV1. In contrast, the clinicopathological features of p-dCJD, such as ataxic gait as an initial symptom, slow progression of neurological symptoms, absence or late occurrence of PSWC on EEG, or formation of PrP^Sc^ plaques in the brain, are similar to those of sCJD-VV2, −MV2K, or -MV2K + C. These similarities raised the possibility that np-dCJD might be caused by infection with sCJD-MM1/MV1, whereas p-dCJD might be caused by infection with sCJD-VV2, −MV2K, or -MV2K + C.

**Figure 4:**
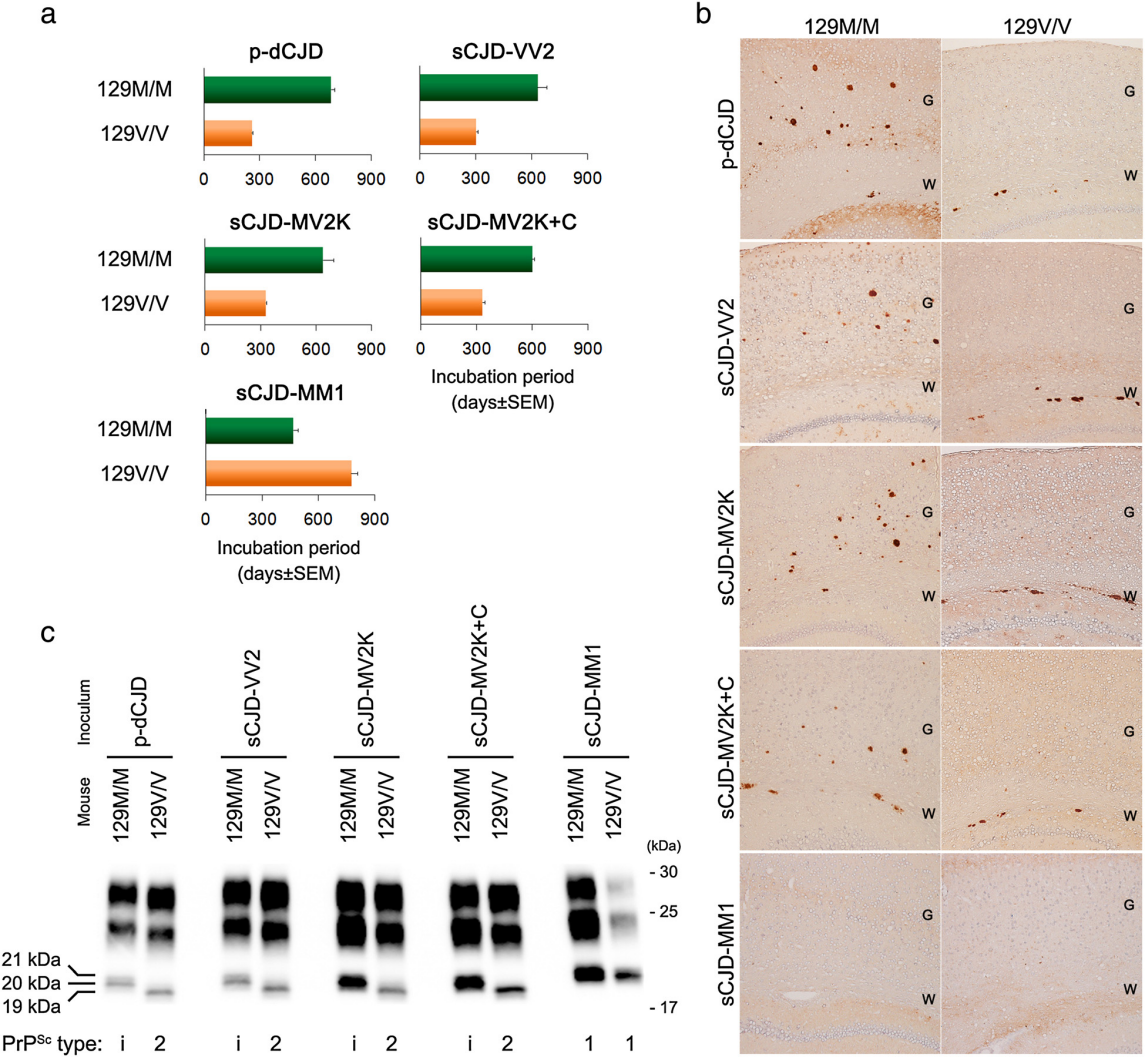
**Transmission of dCJD or sCJD to humanized mice carrying human PrP with either the 129 M/M or 129 V/V genotype. (a)** p-dCJD and sCJD-VV2, −MV2K, or -MV2K + C were identical in the transmissibility to the PrP-humanized mice. **(b)** Immunohistochemical analysis of PrP^Sc^ in the mouse brain revealed that p-dCJD and sCJD-VV2, −MV2K, or -MV2K + C were indistinguishable with regard to the neuropathological phenotypes in the inoculated mice. G, gray matter; W, white matter. **(c)** Westernblot analysis of PrP^Sc^ in the mouse brain showed that p-dCJD and sCJD-VV2, −MV2K, or -MV2K + C were also indistinguishable with regard to the biochemical properties of PrP^Sc^ in the inoculated mice. 129 M/M, knock-in mice expressing human PrP with the 129 M/M genotype; 129 V/V, knock-in mice expressing human PrP with the 129 V/V genotype.

To test this possibility, we examined the transmission properties of the dCJD and sCJD subgroups using humanized mice carrying human PrP with either the 129 M/M or V/V genotype [20,22,23]. In these transmission experiments, p-dCJD and sCJD-VV2, −MV2K, or -MV2K + C were identical in the transmissibility to the PrP-humanized mice (Table 1, Figure 4a) and in the neuropathological and biochemical features in the inoculated mice (Figure 4b, c). By contrast, np-dCJD showed the same transmission properties as sCJD-MM1. In particular, the 129 M/M mice inoculated with sCJD-VV2, −MV2K, or -MV2K + C material showed widespread PrP^Sc^ plaques and type i PrP^Sc^ accumulation similar to the p-dCJD patients, whereas the 129 M/M mice inoculated with sCJD-MM1 material showed diffuse synaptic-type PrP^Sc^ deposition and type 1 PrP^Sc^ accumulation similar to the np-dCJD patients. Thus, these animal models support the hypothesis that the origin of np-dCJD is sCJD-MM1/MV1 and that of p-dCJD is sCJD-VV2, −MV2K, or -MV2K + C. Indeed, the incidence rate of p-dCJD (32%) among total dCJD is close to the sum total of the incidence of sCJD-VV2 (15%), −MV2K (8%), and -MV2K + C (3%) [18].

**Table 1:** Transmission of dCJD or sCJD to PrP-humanized mice

**Inoculum (ID)**	**Incubation period in days ± SEM ( *n */*n***^***0***^**)**^**a**^		
	**129 M/M**		**129 V/V**
	**Tg + Ki-Hu129 M/M**^**b**^	**Ki-Hu129M/M**^**b**^	**Ki-Hu129V/V**^**b**^
	**(9.8×)**^**c**^	**(1×)**	**(1×)**
np-dCJD (GF)	161 ± 5 (5/5)	N.D. ^d^	N.D.
np-dCJD (TC)	208 ± 2 (5/5)	N.D.	N.D.
p-dCJD (KR)	420 ± 10 (5/5)	685 ± 51 (5/5)	259 ± 6 (6/6)
p-dCJD (KD)	398 ± 10 (5/5)	447 ± 51 (6/6)	317 ± 8 (11/11)
sCJD-MM1	175 ± 4 (9/9)	467 ± 24 (8/8)	774 ± 32 (6/6)
sCJD-VV2	505 ± 14 (5/5)	633 ± 49 (6/6)	302 ± 9 (7/7)
sCJD-MV2K	N.D.	638 ± 57 (4/4)	329 ± 3 (4/4)
sCJD-MV2K+C	N.D.	600 ± 22 (6/6)	332 ± 15 (4/4)
129 M/M mouse-passaged sCJD-VV2	N.D.	685 ± 17 (6/6)	309 ± 3 (7/7)

^a^*n*, number of mice positive for PrP accumulation in the immunohistochemical analysis; *n*^0^, number of inoculated mice.

Ki-Hu129M/M, knock-in mice expressing human PrP with the 129 M/M genotype; Ki-Hu129V/V, knock-in mice expressing human PrP with the 129 V/V genotype; Tg + Ki-Hu129M/M, Ki-Hu129M/M crossed with transgenic mice overexpressing human PrP with the 129 M genotype.

^d^N.D., not done.

### Molecular basis of the generation of two distinct subgroups in dCJD

At the molecular level, np-dCJD contains type 1 PrP^Sc^ with the codon 129 M genotype (denoted as M1 PrP^Sc^), whereas p-dCJD contains type i PrP^Sc^ with the codon 129 M genotype (Mi PrP^Sc^) (Table 2). Meanwhile, sCJD-MM1/MV1 contains M1 PrP^Sc^, and sCJD-VV2 contains type 2 PrP^Sc^ with the codon 129 V genotype (V2 PrP^Sc^). Recently, we found that sCJD-MV2K contains Mi PrP^Sc^ and V2 PrP^Sc^, whereas sCJD-MV2K + C also contains type 2 PrP^Sc^ with the codon 129 M genotype and cortical pathology (M2C PrP^Sc^) in addition to Mi PrP^Sc^ and V2 PrP^Sc^ (Table 2) [23]. M2 PrP^Sc^ can be divided into two subgroups based on histopathological phenotypes. M2C PrP^Sc^ causes a predominant cortical pathology in sCJD-MM2C, −MV2C, or -MV2K + C, whereas M2T PrP^Sc^ causes atrophy of thalamic and inferior olivery nuclei in sCJD-MM2T.

**Table 2:** Molecular classification of dCJD and sCJD

**Classification**^**a**^	**Codon 129 genotype**	**PrP**^**Sc **^**type**	**Transmission type**^**b**^	**Original PrP**^**Sc**^	**Existing PrP**^**Sc**^
np-dCJD	M/M	1	M1	M1	M1
p-dCJD	M/M	i ^c^	V2	V2 ^d^	Mi
sCJD-MM1	M/M	1	M1	M1	M1
sCJD-VV2	V/V	2	V2	V2	V2
sCJD-MV2K	M/V	i + 2	V2	V2	Mi + V2
sCJD-MV2K+C	M/V	i + 2	V2	M2C^e^ + V2	M2C + Mi + V2

^a^According to Parchi, 1999 [17]; and Parchi, 2011 [18].

^b^According to Bishop, 2010 [24]; Kobayashi, 2007 [20]; and Kobayashi, 2013 [23].

^c^Intermediate type located between types 1 and 2 PrP^Sc^.

^d^Although not only V2 PrP^Sc^ but also Mi PrP^Sc^ can cause p-dCJD if transmitted to the 129 M/M individuals, the primary origin of Mi PrP^Sc^ is V2 PrP^Sc^ (see text).

^e^M2 PrP^Sc^ can be divided into two subgroups based on histopathological phenotypes. M2C PrP^Sc^ causes a predominant cortical pathology, whereas M2T PrP^Sc^ causes atrophy of thalamic and inferior olivery nuclei.

The generation of M1 PrP^Sc^ in np-dCJD is simply due to the infection with M1 PrP^Sc^ from sCJD-MM1/MV1. On the other hand, the generation of Mi PrP^Sc^ in p-dCJD is rather complicated. Transmission of V2 PrP^Sc^, i.e., sCJD-VV2, to the 129 M/M mice generated Mi PrP^Sc^ (Figure 4c) [20]. Similarly, transmission of Mi PrP^Sc^, i.e., p-dCJD, to the 129 M/M mice also generated Mi PrP^Sc^. Therefore, both V2 PrP^Sc^ and Mi PrP^Sc^ can generate Mi PrP^Sc^ if transmitted to individuals with the 129 M/M genotype. Indeed, transmission of sCJD-MV2K containing Mi PrP^Sc^ and V2 PrP^Sc^ to the 129 M/M mice also generated Mi PrP^Sc^ (Figure 4c). Meanwhile, sCJD-MV2K + C contains M2C PrP^Sc^ besides Mi PrP^Sc^ and V2 PrP^Sc^ (Table 2). However, M2C PrP^Sc^ lacks or has very low infectivity and does not affect the transmission properties of the coexisting PrP^Sc^[23]. Therefore, the transmission of sCJD-MV2K + C to the 129 M/M mice can also result in the generation of Mi PrP^Sc^ (Figure 4c). Taken together, Mi PrP^Sc^ in p-dCJD is generated by infection with Mi PrP^Sc^ and/or V2 PrP^Sc^ from sCJD-VV2, −MV2K, or -MV2K + C. It is noteworthy that Mi PrP^Sc^ can be observed in the 129 M/M mice inoculated with V2 PrP^Sc^ but not in sCJD patients with the 129 M/M genotype, suggesting that Mi PrP^Sc^ in sCJD-MV2K or -MV2K + C is also generated by V2 PrP^Sc^ seed-dependent conversion but not by spontaneous conversion of the 129 M PrP^C^. Therefore, the primary origin of Mi PrP^Sc^ is V2 PrP^Sc^. This can account for the similarities in transmission properties between Mi PrP^Sc^ and V2 PrP^Sc^. Thus, M1 PrP^Sc^ in np-dCJD and Mi PrP^Sc^ in p-dCJD are completely different with regard to the neuropathological phenotypes, biochemical features, and transmission properties, reflecting their distinct PrP^Sc^ origins. In contrast to M1 PrP^Sc^, which is the most common PrP^Sc^ observed in sCJD patients with the 129 M/M genotype, Mi PrP^Sc^ has never been observed in sCJD patients with the 129 M/M genotype. Therefore, the detection of Mi PrP^Sc^ can be sound evidence of iatrogenic infection in individuals with the 129 M/M genotype and would contribute to reliable surveillance of iatrogenic cases such as p-dCJD.

**Figure 5:**
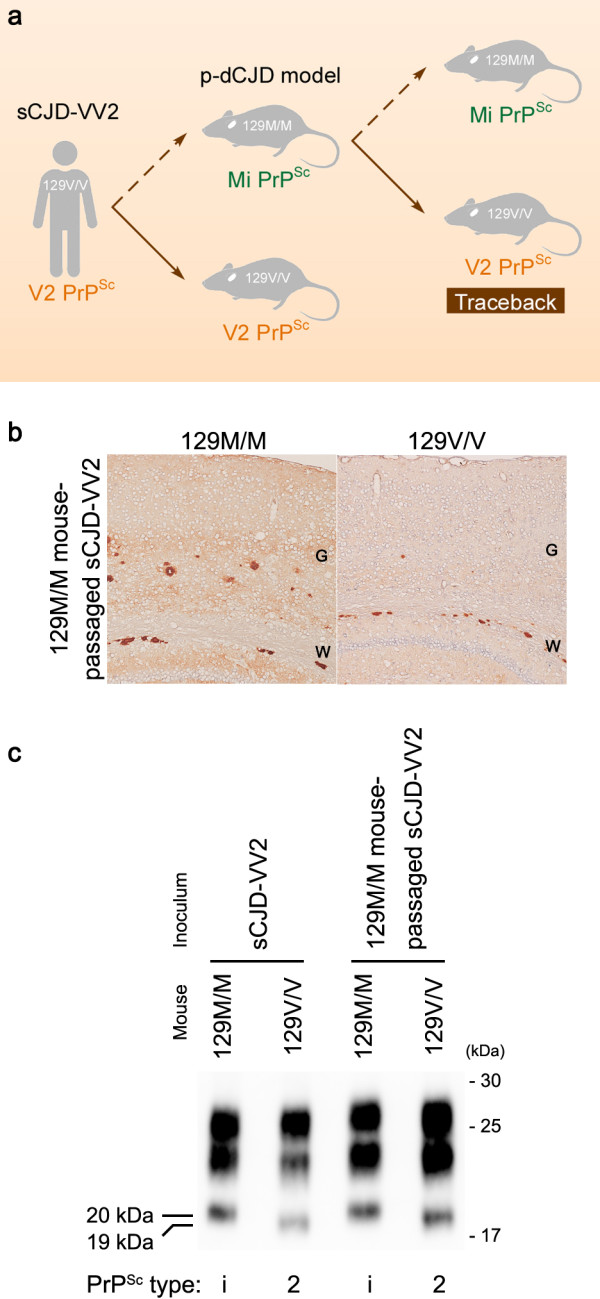
**Modeling of p-dCJD and traceback phenomenon. (a)** Schematic diagram of the modeling study. The 129 V/V mice were highly susceptible to the 129 M/M mouse-passaged sCJD-VV2, i.e., Mi PrP^Sc^, despite their incompatible codon 129 genotypes. Moreover, the altered neuropathological and biochemical phenotypes in the primary passage in the 129 M/M mice reverted to the original ones in the secondary passage in the 129 V/V mice. This is because the origin of Mi PrP^Sc^ is V2 PrP^Sc^. This phenomenon has been designated as traceback [20,26]. **(b)** Immunohistochemical analysis of PrP^Sc^ in the brains from the PrP-humanized mice infected with the 129 M/M mouse-passaged sCJD-VV2. G, gray matter; W, white matter. **(c)** Westernblot analysis of PrP^Sc^ in the brains from the PrP-humanized mice infected with sCJD-VV2 or the 129 M/M mouse-passaged sCJD-VV2. 129 M/M, knock-in mice expressing human PrP with the 129 M/M genotype; 129 V/V, knock-in mice expressing human PrP with the 129 V/V genotype.

To verify experimentally that Mi PrP^Sc^ originates from V2 PrP^Sc^ and its transmission properties are identical to those of the parental V2 PrP^Sc^, we performed a modeling study using PrP-humanized mice (Figure 5a) [25]. As described above, the 129 M/M mice inoculated with V2 PrP^Sc^ showed widespread PrP^Sc^ plaques and Mi PrP^Sc^ accumulation in the brain as an experimental model of p-dCJD. We then inoculated the Mi PrP^Sc^ from these mice into other PrP-humanized mice with either the 129 M/M or V/V genotype. This secondary passage revealed that the transmission properties of the Mi PrP^Sc^, i.e., 129 M/M mouse-passaged sCJD-VV2, are identical to those of the parental V2 PrP^Sc^. In particular, although the incompatibility of the codon 129 genotypes between host and inoculum usually results in a prolonged incubation period [20], the 129 V/V mice inoculated with the Mi PrP^Sc^ showed a shorter incubation period compared with the 129 M/M mice (Table 1). Moreover, the altered neuropathological phenotype and biochemical properties at the primary passage in the 129 M/M mice reverted to the original ones in the secondary passage in the 129 V/V mice (Figure 5b, c). Thus, this modeling study shows that (i) V2 PrP^Sc^ infection in a host with the incompatible codon 129 M/M genotype generates an unusual PrP^Sc^ with altered conformational properties, i.e., Mi PrP^Sc^, (ii) the emerging Mi PrP^Sc^ retains the memory of the parental V2 PrP^Sc^ within its conformational properties, and (iii) the parental V2 PrP^Sc^ re-emerges and proliferates rapidly if the Mi PrP^Sc^ is transmitted to the original host with the codon 129 V/V genotype. This phenomenon, designated as traceback, can be a useful tool to identify the origin of PrP^Sc^ infection if atypical PrP^Sc^ emerges in the future [20,22,26].

### Remaining problems

Our transmission studies resolved the complicated pathogenesis of dCJD. However, they have also revealed several issues surrounding dCJD that need to be addressed in the future.

First, the numbers of p-dCJD patients may increase in the future. The experimental p-dCJD model, i.e., the 129 M/M mice inoculated with Mi PrP^Sc^ and/or V2 PrP^Sc^ from sCJD-VV2, −MV2K, or -MV2K + C, showed a longer incubation period compared with the np-dCJD model, i.e., the 129 M/M mice inoculated with M1 PrP^Sc^ from sCJD-MM1 (Table 1). This raises the concern that additional p-dCJD patients, who are presenting still at the subclinical stage, may emerge after a longer incubation period in the future. Although the numbers of patients with newly developed dCJD have dropped off, continuous surveillance will be required to find remaining p-dCJD cases.

Second, the potential risks of secondary infection from dCJD, particularly from p-dCJD, may be considerable. As described above, the transmission studies raise a concern about the existence of subclinical p-dCJD patients. dCJD patients may undergo more than one neurosurgical operation due to their underlying diseases (the primary disease for which the neurosurgery was performed) [4]. In addition, p-dCJD patients may be more frequently autopsied because the clinical features of p-dCJD are atypical compared with those of classical sCJD [11]. These facts suggest that there may be considerable risk of secondary infection from p-dCJD patients. Individuals with the 129 V/V genotype may be more vulnerable to the infection with Mi PrP^Sc^ from p-dCJD, as suggested by the fact that the 129 V/V mice were highly susceptible to Mi PrP^Sc^ in the transmission study (Table 1). Additionally, 129 M/M individuals may be also affected after a prolonged incubation period, as suggested by the high attack rate (100%) of the 129 M/M mice inoculated with Mi PrP^Sc^. Therefore, secondary infection from p-dCJD can occur regardless of the codon 129 genotype. Comprehensive analysis of the distribution of PrP^Sc^ in the peripheral tissues of p-dCJD patients will be also required to assess the potential risks of secondary infection.

Finally, the efficacy of the current PrP^Sc^ decontamination procedures against Mi PrP^Sc^ needs to be tested in the future. Mi PrP^Sc^ in p-dCJD and M1 PrP^Sc^ in np-dCJD differ in the sizes of the proteinase K-resistant core, suggesting their conformational differences. Moreover, their parental PrP^Sc^ strains are also different. Different PrP^Sc^ strains can show different thermostability [27,28] and different susceptibility to the decontamination procedures [29]. To prevent the spread of secondary infection from dCJD patients to medical workers or other patients, adequate decontamination and disinfection of the instruments used for neurosurgery or autopsy are essential. However, the current PrP^Sc^ decontamination procedures were developed using scrapie isolates and tested using CJD isolates other than p-dCJD [30-32]. Therefore, further studies using Mi PrP^Sc^ will be needed to assess the effectiveness of the current procedures. For this purpose, sensitive detection systems for Mi PrP^Sc^ are also prerequisite to evaluating quantitatively the reduction of infectivity after the decontamination procedures. Real-time quaking-induced conversion [33,34], protein misfolding cyclic amplification [35-39], or transgenic mice overexpressing human PrP with the 129 V genotype [20] might be useful to detect the reduced infectivity of Mi PrP^Sc^ at high sensitivity. Using such sensitive detection systems, effective decontamination procedures for Mi PrP^Sc^ can be established in the future.

### Concluding remarks

Recent progress in the study of the pathogenesis of dCJD has revealed that the two distinct subgroups of dCJD are caused by infection with different PrP^Sc^ strains of sCJD, i.e., np-dCJD caused by M1 PrP^Sc^ from sCJD-MM1/MV1 and p-dCJD caused by Mi PrP^Sc^ and/or V2 PrP^Sc^ from sCJD-VV2, −MV2K, or -MV2K + C. Studies have also revealed previously unrecognized problems such as the considerable risks of secondary infection from dCJD, particularly from p-dCJD. To prevent secondary infection from p-dCJD, the effectiveness of the current decontamination procedures should be tested urgently using sensitive Mi PrP^Sc^ detection systems.

## Competing interest

The authors declare that they have no competing interest.

## References

[B1] Prusiner SB Scott MR DeArmond SJ Cohen FE Prion protein biology Cell 1998 2 337 348 10.1016/S0092-8674(00)81163-0 9590169

[B2] Brown P Brandel JP Sato T Nakamura Y MacKenzie J Will RG Ladogana A Pocchiari M Leschek EW Schonberger LB Iatrogenic Creutzfeldt-Jakob disease, final assessment Emerg Infect Dis 2012 2 901 907 10.3201/eid1806.120116 22607808 PMC3358170

[B3] Hamaguchi T Sakai K Noguchi-Shinohara M Nozaki I Takumi I Sanjo N Sadakane A Nakamura Y Kitamoto T Saito N Mizusawa H Yamada M Insight into the frequent occurrence of dura mater graft-associated Creutzfeldt-Jakob disease in Japan J Neurol Neurosurg Psychiatry 2013 2 1171 1175 10.1136/jnnp-2012-304850 23595947

[B4] Nakamura Y Uehara R Watanabe M Sadakane A Yamada M Mizusawa H Maddox R Sejvar MPH J Belay E Schonberger L Update: Creutzfeldt-Jakob disease associated with cadaveric dura mater grafts - Japan, 1978–2008 MMWR Morb Mortal Wkly Rep 2008 2 1152 115418946463

[B5] Nozaki I Hamaguchi T Sanjo N Noguchi-Shinohara M Sakai K Nakamura Y Sato T Kitamoto T Mizusawa H Moriwaka F Shiga Y Kuroiwa Y Nishizawa M Kuzuhara S Inuzuka T Takeda M Kuroda S Abe K Murai H Murayama S Tateishi J Takumi I Shirabe S Harada M Sadakane A Yamada M Prospective 10-year surveillance of human prion diseases in Japan Brain 2010 2 3043 3057 10.1093/brain/awq216 20855418

[B6] Shimizu S Hoshi K Muramoto T Homma M Ironside JW Kuzuhara S Sato T Yamamoto T Kitamoto T Creutzfeldt-Jakob disease with florid-type plaques after cadaveric dura mater grafting Arch Neurol 1999 2 357 362 10.1001/archneur.56.3.357 10190828

[B7] Hoshi K Yoshino H Urata J Nakamura Y Yanagawa H Sato T Creutzfeldt-Jakob disease associated with cadaveric dura mater grafts in Japan Neurology 2000 2 718 721 10.1212/WNL.55.5.718 10980745

[B8] Mochizuki Y Mizutani T Tajiri N Oinuma T Nemoto N Kakimi S Kitamoto T Creutzfeldt-Jakob disease with florid plaques after cadaveric dura mater graft Neuropathology 2003 2 136 140 10.1046/j.1440-1789.2003.00489.x 12777102

[B9] Kretzschmar HA Sethi S Földvári Z Windl O Querner V Zerr I Poser S Iatrogenic Creutzfeldt-Jakob disease with florid plaques Brain Pathol 2003 2 245 249 12946015 10.1111/j.1750-3639.2003.tb00025.x PMC8095897

[B10] Satoh K Muramoto T Tanaka T Kitamoto N Ironside JW Nagashima K Yamada M Sato T Mohri S Kitamoto T Association of an 11–12kDa protease-resistant prion protein fragment with subtypes of dura graft-associated Creutzfeldt-Jakob disease and other prion diseases J Gen Virol 2003 2 2885 2893 10.1099/vir.0.19236-0 13679624

[B11] Noguchi-Shinohara M Hamaguchi T Kitamoto T Sato T Nakamura Y Mizusawa H Yamada M Clinical features and diagnosis of dura mater graft associated Creutzfeldt-Jakob disease Neurology 2007 2 360 367 10.1212/01.wnl.0000266624.63387.4a 17646628

[B12] Yamada M Noguchi-Shinohara M Hamaguchi T Nozaki I Kitamoto T Sato T Nakamura Y Mizusawa H Dura mater graft-associated Creutzfeldt-Jakob disease in Japan: clinicopathological and molecular characterization of the two distinct subtypes Neuropathology 2009 2 609 618 10.1111/j.1440-1789.2008.00987.x 19659940

[B13] Lane KL Brown P Howell DN Chain BJ Hulette CM Burger PC DeArmond SJ Creutzfeldt-Jakob disease in a pregnant woman with an implanted dura mater graft Neurosurgery 1994 2 737 740 10.1227/00006123-199404000-00026 8008176

[B14] Kopp N Streichenberger N Deslys JP Laplanche JL Chazot G Creutzfeldt-Jakob disease in a 52-year-old woman with florid plaques Lancet 1996 2 1239 1240 8898048 10.1016/s0140-6736(05)65510-9

[B15] Takashima S Tateishi J Taguchi Y Inoue H Creutzfeldt-Jakob disease with florid plaques after cadaveric dural graft in a Japanese woman Lancet 1997 2 865 866 9310612 10.1016/S0140-6736(05)62035-1

[B16] Kimura K Nonaka A Tashiro H Yaginuma M Shimokawa R Okeda R Yamada M Atypical form of dura graft associated Creutzfeldt-Jakob disease: report of a postmortem case with review of the literature J Neurol Neurosurg Psychiatry 2001 2 696 699 10.1136/jnnp.70.5.696 11309472 PMC1737365

[B17] Parchi P Giese A Capellari S Brown P Schulz-Schaeffer W Windl O Zerr I Budka H Kopp N Piccardo P Poser S Rojiani A Streichemberger N Julien J Vital C Ghetti B Gambetti P Kretzschmar H Classification of sporadic Creutzfeldt-Jakob disease based on molecular and phenotypic analysis of 300 subjects Ann Neurol 1999 2 224 233 10.1002/1531-8249(199908)46:2<224::AID-ANA12>3.0.CO;2-W 10443888

[B18] Parchi P Strammiello R Giese A Kretzschmar H Phenotypic variability of sporadic human prion disease and its molecular basis: past, present, and future Acta Neuropathol 2011 2 91 112 10.1007/s00401-010-0779-6 21107851

[B19] Parchi P Zou W Wang W Brown P Capellari S Ghetti B Kopp N Schulz-Schaeffer WJ Kretzschmar HA Head MW Ironside JW Gambetti P Chen SG Genetic influence on the structural variations of the abnormal prion protein Proc Natl Acad Sci USA 2000 2 10168 10172 10.1073/pnas.97.18.10168 10963679 PMC27779

[B20] Kobayashi A Asano M Mohri S Kitamoto T Cross-sequence transmission of sporadic Creutzfeldt-Jakob disease creates a new prion strain J Biol Chem 2007 2 30022 30028 10.1074/jbc.M704597200 17709374

[B21] Parchi P Strammiello R Notari S Giese A Langeveld JP Ladogana A Zerr I Roncaroli F Cras P Ghetti B Pocchiari M Kretzschmar H Capellari S Incidence and spectrum of sporadic Creutzfeldt-Jakob disease variants with mixed phenotype and co-occurrence of PrP^Sc^ types: an updated classification Acta Neuropathol 2009 2 659 671 10.1007/s00401-009-0585-1 19718500 PMC2773124

[B22] Kobayashi A Asano M Mohri S Kitamoto T A traceback phenomenon can reveal the origin of prion infection Neuropathology 2009 2 619 624 10.1111/j.1440-1789.2008.00973.x 19659941

[B23] Kobayashi A Iwasaki Y Otsuka H Yamada M Yoshida M Matsuura Y Mohri S Kitamoto T Deciphering the pathogenesis of sporadic Creutzfeldt-Jakob disease with codon 129M/V and type 2 abnormal prion protein Acta Neuropathol Commun 2013 2 74 10.1186/2051-5960-1-74 24252157 PMC3833290

[B24] Bishop MT Will RG Manson JC Defining sporadic Creutzfeldt-Jakob disease strains and their transmission properties Proc Natl Acad Sci USA 2010 2 12005 12010 10.1073/pnas.1004688107 20547859 PMC2900653

[B25] Kobayashi A Sakuma N Matsuura Y Mohri S Aguzzi A Kitamoto T Experimental verification of a traceback phenomenon in prion infection J Virol 2010 2 3230 3238 10.1128/JVI.02387-09 20089646 PMC2838106

[B26] Asano M Mohri S Ironside JW Ito M Tamaoki N Kitamoto T vCJD prion acquires altered virulence through trans-species infection Biochem Biophys Res Commun 2006 2 293 299 10.1016/j.bbrc.2006.01.149 16480953

[B27] Taylor DM Fraser H McConnell I Brown DA Brown KL Lamza KA Smith GR Decontamination studies with the agents of bovine spongiform encephalopathy and scrapie Arch Virol 1994 2 313 326 10.1007/BF01310794 7832638

[B28] Rutala WA Weber DJ Creutzfeldt-Jakob disease: recommendations for disinfection and sterilization Clin Infect Dis 2001 2 1348 1356 10.1086/319997 11303271

[B29] Rogez-Kreuz C Yousfi R Soufflet C Quadrio I Yan ZX Huyot V Aubenque C Destrez P Roth K Roberts C Favero M Clayette P Inactivation of animal and human prions by hydrogen peroxide gas plasma sterilization Infect Control Hosp Epidemiol 2009 2 769 777 10.1086/598342 19563265

[B30] Brown P Gibbs CJ Jr Amyx HL Kingsbury DT Rohwer RG Sulima MP Gajdusek DC Chemical disinfection of Creutzfeldt-Jakob disease virus N Engl J Med 1982 2 1279 1282 10.1056/NEJM198205273062107 7040968

[B31] Taguchi F Tamai Y Uchida K Kitajima R Kojima H Kawaguchi T Ohtani Y Miura S Proposal for a procedure for complete inactivation of the Creutzfeldt-Jakob disease agent Arch Virol 1991 2 297 301 10.1007/BF01310679 1877889

[B32] Tateishi J Tashima T Kitamoto T Practical methods for chemical inactivation of Creutzfeldt-Jakob disease pathogen Microbiol Immunol 1991 2 163 166 10.1111/j.1348-0421.1991.tb01544.x 1909414

[B33] Wilham JM Orrú CD Bessen RA Atarashi R Sano K Race B Meade-White KD Taubner LM Timmes A Caughey B Rapid end-point quantitation of prion seeding activity with sensitivity comparable to bioassays PLoS Pathog 2010 2 e1001217 10.1371/journal.ppat.1001217 21152012 PMC2996325

[B34] Atarashi R Satoh K Sano K Fuse T Yamaguchi N Ishibashi D Matsubara T Nakagaki T Yamanaka H Shirabe S Yamada M Mizusawa H Kitamoto T Klug G McGlade A Collins SJ Nishida N Ultrasensitive human prion detection in cerebrospinal fluid by real-time quaking-induced conversion Nat Med 2011 2 175 178 10.1038/nm.2294 21278748

[B35] Saborio GP Permanne B Soto C Sensitive detection of pathological prion protein by cyclic amplification of protein misfolding Nature 2001 2 810 813 10.1038/35081095 11459061

[B36] Suyama K Yoshioka M Akagawa M Murayama Y Horii H Takata M Yokoyama T Mohri S Assessment of prion inactivation by fenton reaction using protein misfolding cyclic amplification and bioassay Biosci Biotechnol Biochem 2007 2 2069 2071 10.1271/bbb.70085 17690456

[B37] Yoshioka M Murayama Y Miwa T Miura K Takata M Yokoyama T Nishizawa K Mohri S Assessment of prion inactivation by combined use of Bacillus-derived protease and SDS Biosci Biotechnol Biochem 2007 2 2565 2568 10.1271/bbb.70257 17928684

[B38] Beekes M Lemmer K Thomzig A Joncic M Tintelnot K Mielke M Fast, broad-range disinfection of bacteria, fungi, viruses and prions J Gen Virol 2010 2 580 589 10.1099/vir.0.016337-0 19864502

[B39] Takeuchi A Komiya M Kitamoto T Morita M Deduction of the evaluation limit and termination timing of multi-round protein misfolding cyclic amplification from a titration curve Microbiol Immunol 2011 2 502 509 10.1111/j.1348-0421.2011.00340.x 21443616

